# Genomic and phenotypic characterization of multidrug-resistant *Salmonella enterica* serovar Reading isolates involved in a turkey-associated foodborne outbreak

**DOI:** 10.3389/fmicb.2023.1304029

**Published:** 2024-01-18

**Authors:** Sathesh K. Sivasankaran, Bradley L. Bearson, Julian M. Trachsel, Daniel W. Nielsen, Torey Looft, Shawn M. D. Bearson

**Affiliations:** ^1^USDA, ARS, National Animal Disease Center, Food Safety and Enteric Pathogens, Ames, IA, United States; ^2^Genome Informatics Facility, Iowa State University, Ames, IA, United States; ^3^Agroecosystems Management Research Unit, USDA, ARS, National Laboratory for Agriculture and the Environment, Ames, IA, United States; ^4^ARS Research Participation Program, Oak Ridge Institute for Science and Education (ORISE), Oak Ridge, TN, United States

**Keywords:** *Salmonella enterica* serovar Reading, foodborne outbreak, turkeys, genomic comparison, *cirA*, colicin, cefiderocol, glucuronide utilization

## Abstract

*Salmonella* is a global bacterial foodborne pathogen associated with a variety of contaminated food products. Poultry products are a common source of *Salmonella*-associated foodborne illness, and an estimated 7% of human illnesses in the United States are attributed to turkey products. From November 2017 to March 2019, the Centers for Disease Control and Prevention reported a turkey-associated outbreak of multidrug-resistant (MDR; resistant to ≥3 antimicrobial classes) *Salmonella enterica* serovar Reading (*S*. Reading) linked to 358 human infections in 42 US states and Canada. Since *S*. Reading was seldom linked to human illness prior to this outbreak, the current study compared genomic sequences of *S*. Reading isolates prior to the outbreak (pre-outbreak) to isolates identified during the outbreak period, focusing on genes that were different between the two groups but common within a group. Following whole-genome sequence analysis of five pre-outbreak and five outbreak-associated turkey/turkey product isolates of *S*. Reading, 37 genes located within two distinct chromosomal regions were identified only in the pre-outbreak isolates: (1) an ~5 kb region containing four protein-coding genes including *uidA* which encodes beta-glucuronidase, *pgdA* encoding peptidoglycan deacetylase, and two hypothetical proteins and (2) an ~28 kb region comprised of 32 phage-like genes and the *xerC* gene, which encodes tyrosine recombinase (frequently associated with phage genes). The five outbreak isolates also had a deletional event within the *cirA* gene, introducing a translational frame shift and premature stop codon. The *cirA* gene encodes a protein with dual receptor functions: a siderophore receptor for transport of dihydroxybenzoylserine as well as a colicin Ia/b receptor. Significant differences for the identified genetic variations were also detected in 75 *S*. Reading human isolates. Of the 41 *S*. Reading isolates collected before or in 2017, 81 and 90% of the isolates contained the *uidA* and *pgdA* genes, respectively, but only 24% of the isolates collected after 2017 harbored the *uidA* and *pgdA* genes. The truncation event within the *cirA* gene was also significantly higher in isolates collected after 2017 (74%) compared to before or in 2017 (5%). Phenotypic analysis of the *S*. Reading isolates for colicin and cefiderocol sensitivities (CirA) and β-methyl-D-glucuronic acid utilization (UidA and accessory proteins) supported the genomic data. Overall, a similar genome reduction pattern was generally observed in both the turkey and human isolates of *S*. Reading during the outbreak period, and the genetic differences were present in genes that could potentially promote pathogen dissemination due to variation in *Salmonella* colonization, fitness, and/or virulence.

## 1 Introduction

*Salmonella enterica* subspecies *enterica* is a major human foodborne pathogen that can subclinically colonize food-producing animals, resulting in unrecognized animal carriage and subsequent contamination of animal food products. From 2009 to 2021, the Centers for Disease Control and Prevention (CDC) documented 1,712 foodborne outbreaks involving *Salmonella* in the United States (U.S.), with 47 of the outbreaks associated with turkey products (CDC, 2023). From November 2017 to March 2019, a turkey-associated outbreak of multidrug-resistant (MDR; resistant to ≥3 antimicrobial classes) *Salmonella enterica* serovar Reading (*S*. Reading) was linked to 358 human infections and one death across 42 US states and Canada (CDC, 2019; PHAC, 2020). Traceback investigations did not identify an individual brand, company, or source of turkey product responsible for the outbreak, prompting the first industry group engagement by the CDC and USDA Food Safety Inspection Service (FSIS) to reduce *Salmonella* in their products rather than direct interaction with a specific company (Hassan et al., 2019). Ultimately, four recalls were issued for products containing outbreak *S*. Reading, including two ground turkey products and two raw turkey pet foods.

*Salmonella enterica* serovar Reading can colonize turkeys (Hird et al., 1993; Poppe et al., 1995; Anderson et al., 2010; Ashcraft et al., 2021), but historically has not been associated with human foodborne illness (CDC, 2018). Evidence that outbreak *S*. Reading was present in birds from turkey production facilities nationwide supported the determination of *S*. Reading in turkey products as the outbreak etiological agent and source (Hassan et al., 2019). Since *S*. Reading was seldom linked to human illness prior to this outbreak, the current study compared genomic sequences of *S*. Reading isolates prior to the outbreak (pre-outbreak) to *S*. Reading isolated during the outbreak period to identify genes that were different between the two groups but common within a group, thereby potentially identifying genetic alterations contributing to the outbreak status of *S*. Reading. The study identified genetic variations between the pre-outbreak and outbreak isolates of *S*. Reading in both turkeys/turkey products and human clinical infections. Phenotypic analyses confirmed certain genetic differences, suggesting genome alterations in *S*. Reading in the outbreak period could have contributed to variation in *Salmonella* colonization, fitness, or virulence in the turkey and/or human host.

## 2 Materials and methods

### 2.1 *Salmonella enterica* serovar Reading isolates and growth conditions

*Salmonella enterica* serovar Reading isolates listed in Supplementary Table S1 from turkeys/turkey products or humans were received from the USDA, FSIS (Supplementary Table S1), and the CDC (Supplementary Tables S2, S3), respectively, and stocked at −80°C in 15% glycerol and 0.9% sodium chloride. Frozen isolates were streaked for isolation onto Luria-Bertani (LB; Lennox) agar (Sigma-Aldrich, St. Louis, MO, United States) and incubated at 37°C overnight. An individual colony was selected and inoculated into 3 mL of LB broth (Thermo Fisher Scientific, Wilmington, DE, United States) at 37°C overnight with shaking for further analysis in the assays described below.

To facilitate the construction of nalidixic acid-resistant *S.* Reading isolates by lambda red recombineering (for selection in the turkey trial described below), SX439, SX440, SX441, SX442, SX443, SX445, and SX448 were transformed by electroporation with pKD46 (Datsenko and Wanner, 2000); isolates SX444, SX446, and SX447 were transformed with pKD46-Gm because they were already resistant to ampicillin (Doublet et al., 2008). Primers oBBI 535 (5′ P GCCCGTGTCGTTGGTGACGTAATC 3′) and oBBI 536 (5′ CGACGTGCGCTGGATAAAACGCTG 3′), which bind to *gyrA* and *rcsC*, respectively, were used to PCR amplify the *gyrA* gene containing a point mutation rendering nalidixic acid resistance from *Salmonella enterica* serovar Typhimurium strain BSX 8 (χ4232). The *S.* Reading isolates containing pKD46 or pKD46-Gm were transformed with a purified PCR product of oBBI 535/oBBI 536 following arabinose induction of the lambda *bet*, *gam*, and *exo* genes. The transformed bacterial cells were plated on LB agar containing nalidixic acid (30 μg/mL) to select for nalidixic acid resistance. The nalidixic acid-resistant *S.* Reading isolates were cured with temperature-sensitive pKD46 or pKD46-Gm, followed by stocking at −80°C in 15% glycerol and 0.9% sodium chloride as the corresponding SX449-SX458 to be used for further characterization. Individual colonies were selected and inoculated into 3 mL of Luria-Bertani broth (LB, Lennox; Invitrogen, Waltham, MA, United States) at 37°C overnight with shaking for further analysis.

### 2.2 Genomic DNA extraction and sequencing

Overnight *Salmonella* cultures were centrifuged for 20 min at 3,000 × *g*. The supernatants were discarded, and the bacterial pellets were resuspended in 400 μL of phosphate-buffered saline. DNA isolation from 100 μL of the bacterial resuspension was performed using the High Pure PCR Template Preparation Kit (Roche Applied Science, Indianapolis, IN, United States) per the manufacturer’s instructions. The quality and quantity of DNA were measured on the Qubit 4 Fluorometer using the Qubit™ dsDNA Broad Range Assay Kit (Invitrogen, Waltham, MA, United States). Long- and short-read genome/nucleotide sequences for 10 FSIS isolates were generated on an Oxford Nanopore GridION X5 (Oxford Nanopore Technologies, Oxford, United Kingdom) and an Illumina MiSeq (San Diego, CA, United States) at the National Animal Disease Center (NADC) Genomics Facility (Ames, IA, United States; Supplementary Table S4), respectively. Whole-genome sequencing (WGS) libraries for long-read sequencing were prepared with the SQK-RBK004 rapid barcoding kit (Nanopore, Oxford, United Kingdom) according to the manufacturer’s instructions. WGS libraries for short-read sequencing were generated using the Nextera DNA Flex Library Prep and Indexes kits (Illumina, San Diego, CA, United States) and sequenced using the MiSeq reagent kit v3 (600-cycle), yielding 2 × 300-bp paired-end reads on the MiSeq platform.

### 2.3 Pan-genome and single nucleotide polymorphism analyses

#### 2.3.1 FSIS isolates

Illumina short reads generated by FSIS were downloaded from the Sequence Read Archive (SRA-NCBI; Supplementary Table S1) using the SRA Toolkit (NCBI, 2023). Reads for in-house and downloaded sequences were assembled using hybrid assembler Unicycler v0.4 (Wick et al., 2017) with default parameters. The completeness and contamination of the assembled genomes were checked using checkM (Parks et al., 2015). Genomes were annotated using Prokka (Seemann, 2014) with default parameters. The presence of plasmid replicons in each genome was tested using ABRicate with the PlasmidFinder database (Carattoli et al., 2014; Seemann, 2020a). Contigs with replicons were identified as plasmid sequences if they circularized and were not identified as the bacterial chromosome. Pan-genome analysis was run with Roary (Page et al., 2015). Both substitutions (single nucleotide polymorphism; SNP) and insertions/deletions (indels) were analyzed using Snippy (Seemann, 2020b) with SX441 as a reference. Multiple sequence alignments of the CirA protein were generated using the constraint-based Multiple Alignment Tool (COBALT, 2007) with default parameters.

#### 2.3.2 CDC isolates

Illumina raw reads for 64 CDC *S*. Reading isolates were downloaded from SRA-NCBI using the SRA toolkit (Supplementary Table S2). Nucleotide sequences for 11 CDC isolates were generated (Supplementary Tables S3, S4) on an Illumina MiSeq as described above. Read assembly, genome annotation, and pan-genome analysis of the CDC isolates were performed as described above. The isolates were divided into two groups based on the year of collection: isolates collected before or in 2017 were placed in group 1, and isolates collected after 2017 were placed in group 2. The statistical significance of the presence/absence of *uidA*, *pgdA* genes, and phage-like regions between groups was analyzed using the Fisher’s exact test in R, as well as the significance of the full length/truncation (mutation described in Table 1) of the *cirA* gene between groups.

**Table 1:** Single nucleotide polymorphisms (SNP) and insertions/deletions (indels) sequence differences between pre-outbreak and outbreak *Salmonella enterica* serovar Reading isolates from turkeys/turkey products.

Position	Type*	Reference	Alternative	Gene	Effect	Gene description
2,14,316	SNP	T	G	*bisC*	missense c.1655 T > G p.Val552Gly	Biotin sulfoxide reductase
2,92,507	SNP	T	A		missense c.17A > T p.Lys6Ile	hypothetical protein
6,06,208	SNP	G	C	*tsaR*	missense c.719G > C p.Gly240Ala	HTH-type transcriptional regulator TsaR
7,15,553	SNP	A	G	*yghU*	missense c.515 T > C p.Ile172Thr	Disulfide-bond oxidoreductase YghU
7,49,131	ins	A	AT			Intergenic region
7,78,397	SNP	C	T	*loiP*	missense c.625G > A p.Gly209Arg	Metalloprotease LoiP
9,20,038	del	TG	T			Intergenic region
11,19,430	SNP	C	T		synonymous c.6198G > A p.Leu2066Leu	hypothetical protein
12,87,895	SNP	G	A		missense c.2471G > A p.Gly824Glu	hypothetical protein
13,72,118	SNP	G	T	*amiA*	missense c.419C > A p.Pro140Gln	N-acetylmuramoyl-L-alanine amidase AmiA
15,32,139	SNP	C	T	*arnT*	synonymous c.564G > A p.Pro188Pro	Undecaprenyl phosphate-alpha-4-amino-4-deoxy-L-arabinose arabinosyl transferase
16,17,730	SNP	G	T	*fruA1*	synonymous c.516G > T p.Ala172Ala	PTS system fructose-specific EIIB’BC component
16,24,618	del	GC	G	*cirA*	frameshift c.680delC p.Pro227fs	Colicin I receptor
19,95,693	SNP	C	A	*yabJ*	missense c.160G > T p.Gly54Cys	2-iminobutanoate/2-iminopropanoate deaminase
24,96,971	SNP	G	A	*ppsA*	missense c.1268C > T p.Ala423Val	Phosphoenolpyruvate synthase
25,42,325	SNP	A	G	*astA*	synonymous c.267 T > C p.Tyr89Tyr	Arginine N-succinyltransferase
30,18,682	SNP	G	A		missense c.346G > A p.Gly116Ser	hypothetical protein
30,87,507	SNP	G	A	*sdhA*	missense c.677C > T p.Ala226Val	Succinate dehydrogenase flavoprotein subunit
37,57,773	SNP	G	A	*guaC*	synonymous c.513C > T p.Ser171Ser	GMP reductase
37,71,328	SNP	C	T	*murG*	missense c.1049G > A p.Ser350Asn	UDP-N-acetylglucosamine--N-acetylmuramyl-(pentapeptide) pyrophosphoryl-undecaprenol N-acetylglucosamine transferase
46,81,066	SNP	T	C	*yidC*	missense c.180A > G p.Ile60Met	Membrane protein insertase YidC

^*^SNP, Single nucleotide polymorphism; ins, insertion; and del, deletion.

#### 2.3.3 Pathogen detection database (NCBI) isolates

The metadata associated with the NCBI Pathogen Detection Database (NCBI, 2016) version PD000000002.2312 was downloaded (10.01.21). The metadata containing *S.* Reading isolates were filtered using the “geo_loc_name,” “epi_type,” “collect_year,” and “create_year” columns in the metadata provided by the NCBI PDD. The isolates from the North American region (United States, Canada, or Mexico in the “geo_loc_name” column) were included only in the analysis. The isolates were excluded if the “collect_year” or “create_year” columns in the metadata were missing. The year in the “create_year” column was considered if the “collect_year” was missing. Using the above-mentioned criteria, a total of 2,461 *S*. Reading isolates from North America [United States (91.4%; 2,250), Canada (7.5%; 184), and Mexico (1.1%; 27)] were selected for the analysis (Supplementary Table S5). Genomes were annotated with Prokka, and core genome alignment was generated using Roary. Categorization and statistical significance were performed as described for the CDC isolates (above).

### 2.4 Colicin sensitivity assay (agar overlay)

Colicin sensitivity was assayed as described (Nedialkova et al., 2014). Briefly, an overnight culture of colicin-producing *Salmonella enterica* serovar Typhimurium strain SL1344 (Spriewald et al., 2015) was diluted to OD_600nm_ = 0.8, 25 μL was spotted in the center of LB agar plates containing 0.5 μg/mL mitomycin C (Sigma-Aldrich) and 100 μM diethylenetriamine pentaacetic acid (DTPA; Sigma-Aldrich), and grown overnight for 20 h at 37°C. To 10 mL of top agar (0.75% agar), 10 μL of an overnight culture of an individual *S*. Reading isolate (tester isolate) was added and gently vortexed. The top agar containing the *S*. Reading isolate was poured onto the plate previously spotted with strain SL1344, incubated for 20 h at 37°C, and the formation of an inhibition zone (halo) around colicin-producing SL1344 was measured to determine the sensitivity of each *S*. Reading isolate.

### 2.5 Cefiderocol disk diffusion assay

*Salmonella* isolates were evaluated for susceptibility to cefiderocol by growing a 1:100 dilution of the overnight culture to O.D._600nm_ = 0.4. The culture was swabbed onto a Mueller-Hinton agar plate, and a single HardyDisks™ Cefiderocol (30 μg, Hardy Diagnostics, Santa Maria, CA, United States) was laid on top of the agar at the center of the petri plate and incubated at 37°C for 18–20 h. The zone of inhibition around the cefiderocol disk was measured to determine antibiotic sensitivity.

### 2.6 Biolog phenotype microarray analysis of β-methyl-D-glucuronic acid

To determine bacterial utilization of glucuronides as a carbon source, phenotype microarray assays (Biolog, Hayward, CA, United States) were performed on the *S.* Reading isolates from FSIS using microplate PM2 with inoculating fluid IF-0. The PM2 microplate was inoculated as described by the manufacturer and incubated at 33°C for 48 h in a Biolog GEN III OmniLog System with automated plate readings for data collection at 15-min intervals. All isolates were assayed twice on separate days. The maximal growth curve value for Biolog phenotype microarray data from well C9 containing β-methyl-D-glucuronic acid was averaged for each isolate, and the phenotype was compared between pre-outbreak and outbreak isolates using an unpaired t-test in GraphPad Prism 5.01 (GraphPad Software, San Diego, CA, United States). A *p* value <0.05 was considered significantly different between the groups.

### 2.7 Turkey challenge study, sample processing, and statistical analysis

One-day-old hybrid jake (male) turkey poults were obtained from a commercial farm and randomly distributed to four ABSL-2 isolation rooms at the National Animal Disease Center (*n* = 24/room). Intestines and yolks from 10 poults tested upon arrival were negative for *Salmonella* using qualitative bacteriology, as previously described (Bearson et al., 2016). On the day of arrival, poults were inoculated by oral gavage with 0.25 mL of 1 × 10^4^ colony-forming units (CFU) of *S*. Reading isolates SX450, SX453, SX456, or SX458 (nalidixic acid-resistant strains of the USDA, FSIS isolates). Throughout the study, poults were fed a turkey poult starter ration and had water available *ad libitum*. At 7- and 35-day post-inoculation (dpi), 10–12 turkeys randomly selected from each group were euthanized using carbon dioxide or barbiturates at the labeled dose (based on weight). From each bird, 1 g of tissue samples from the cecum, spleen, and bursa of Fabricius were aseptically collected for *S.* Reading detection and enumeration, as previously described (Bearson et al., 2016). Tissue samples that were negative by both quantitative and qualitative bacteriology were assigned 0 CFU/g, while those positive only by enrichment were assigned a random value between 2 and 19 CFU/g (limit of detection). The enumeration data was log_10_ transformed, and using GraphPad Prism 5.01, a one-way ANOVA with a Tukey’s multiple comparison test was used to determine statistical significance for tissue colonization between the different isolates. For all statistical tests listed above, significance was set at *p* < 0.05. Procedures involving animals followed protocols approved by the USDA, ARS, and National Animal Disease Center Animal Care and Use Committee in strict accordance with the recommendations in the Guide for the Care and Use of Laboratory Animals by the National Research Council of the National Academies.

## 3 Results

### 3.1 Genomic differences identified between pre-outbreak and outbreak *Salmonella enterica* serovar Reading isolates from turkeys/turkey products

To identify genetic differences between *S*. Reading isolated from turkeys/turkey products during the United States. Outbreak period compared to *S*. Reading isolated before the outbreak, genomic sequencing and analysis were performed on 10 isolates from the FSIS collection (five pre-outbreak isolates and five outbreak isolates). The 10 *S.* Reading isolates were from either turkeys or comminuted turkey products from various states within the United States Genomic sequencing information and antibiotic resistance profiles are detailed in Supplementary Tables S1, S4. Our quality analysis revealed that the assembled, closed genomes (including plasmids) are 100% complete (0.33% contamination with 0% heterogeneity) with a GC content of 52% and a length of ~4.6 Mb. Compared to the pre-outbreak isolates, an ~30 kb reduction was observed in the genome size of the outbreak isolates, even though all 10 isolates have the same pulse field gel electrophoresis (PFGE) pattern (JLGX01.0098). All isolates harbored a ColpVC plasmid, four isolates contained ColRNA1-type plasmids, and two isolates harbored an IncQ1-type plasmid (Supplementary Table S4).

**Figure 1:**
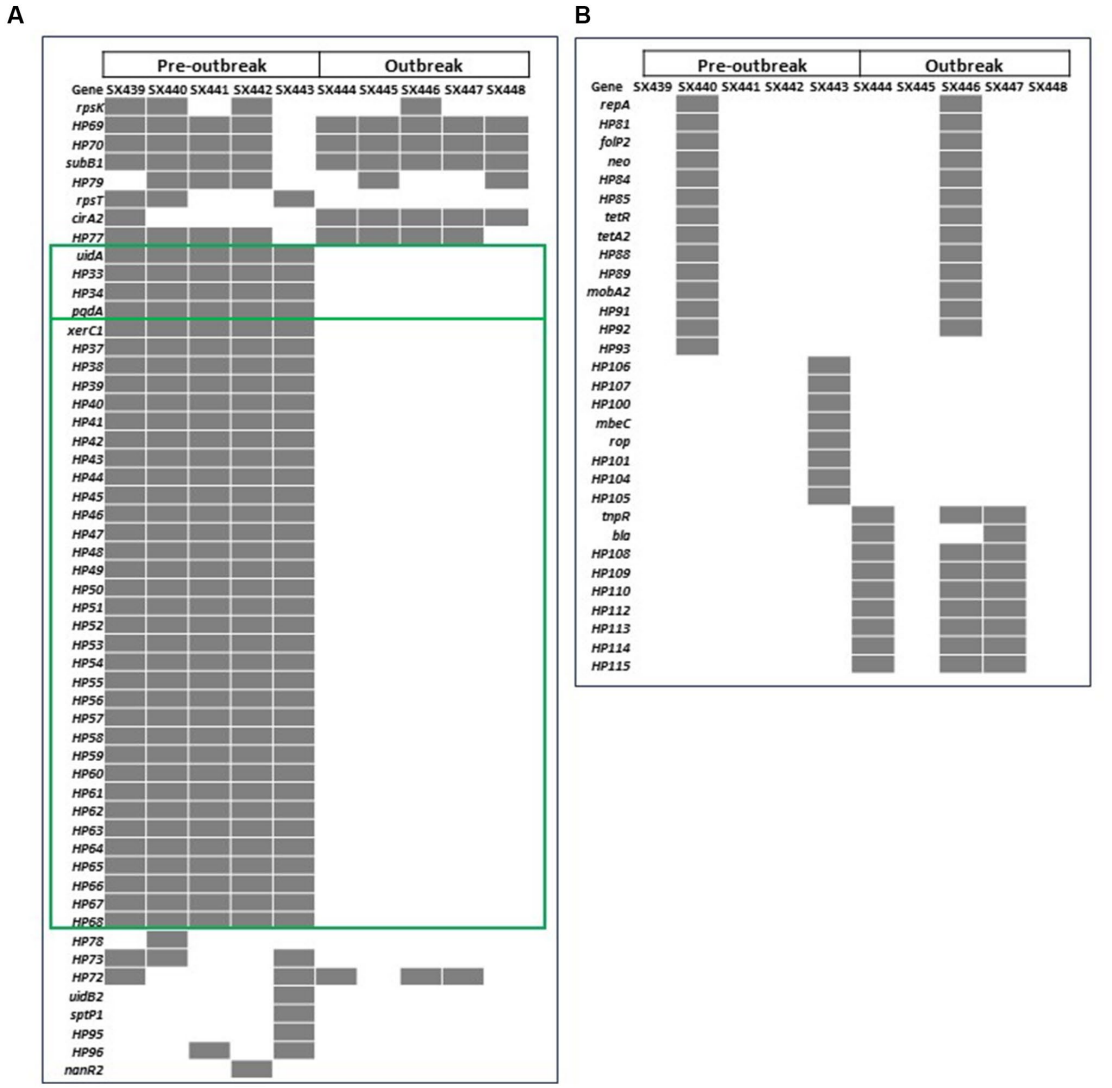
Heatmap of the 84 genes with differences in the presence (gray) and absence (white) in pre-outbreak and outbreak *Salmonella enterica* serovar Reading isolates from FSIS turkeys/turkey products in the **(A)** chromosome and **(B)** plasmids. Green boxes highlight the 37 genes from two genomic locations present in all pre-outbreak isolates and absent in all post-outbreak isolates.

Pan-genome analysis identified a total of 4,456 genes in the 10 isolates combined, of which 4,372 genes (98.1%) were present in all isolates (the core genes). Of the 84 genes (1.9%) missing in at least one of the isolates, 53 genes were chromosomally located, and the remaining 31 genes were detected on a plasmid. With the research objective of identifying genetic difference(s) that may have contributed to the outbreak status of *S*. Reading, further comparative analyses between the pre-outbreak and outbreak isolates focused on the genes that were different between the two outbreak groups but common within a group. Using this criteria, 37 genes located within two distinct chromosomal regions were investigated further (Figure 1; Supplementary Table S6). An ~5.4 kb region containing *uidA* encoding β-glucuronidase, *pgdA* encoding peptidoglycan deacetylase, and two genes encoding hypothetical proteins were present only in the pre-outbreak isolates. A 10-bp sequence (CGCGGGTAAA) duplication flanking the *uidA* region in pre-outbreak isolates was identified; due to the absence of the ~5.4 kb sequence in outbreak isolates, only a single 10-mer was detected, suggesting the occurrence of a deletional event in this area of homology. Also absent in the outbreak isolates was an ~28 kb region comprised of 32 phage-like genes and *xerC*, which encodes tyrosine recombinase. An imperfect duplication of a 20-bp sequence (AAAACGCGCCCGAAGG**C/T**GCG) flanking the phage-like element (located in the intergenic region between *yfiA* and *pheA*) was identified in the pre-outbreak isolates; only a single 20-mer (AAAACGCGCCCGAAGG**C**GCG) was detected in the outbreak isolates, suggesting the phage-like region was lost in a recombinational event. Using the criteria described above, plasmid-encoded gene differences were not observed between the pre-outbreak and outbreak groups.

Snippy analysis detected 21 additional variations between the pre-outbreak and outbreak groups, including 18 SNPs, two deletions, and one insertion (Table 1). The genetic variations between the groups resulted in 13 missense mutations (10 in genes with known function and three in functionally unknown genes) and five synonymous variations. A deletion and an insertion were identified in intergenic regions, while another deletion was identified within the *cirA* gene, which encodes a transporter for dihydroxybenzoylserine (a siderophore and salmochelin/enterochelin breakdown product) as well as a receptor for colicin Ia/b. Analysis of the *cirA* gene and predicted protein sequences for 10 FSIS isolates revealed a cytosine deletion at nucleotide 680 of the *cirA* coding region in all five outbreak isolates that resulted in a translational frameshift with an altered protein sequence beginning at AA227 and truncation of the protein at AA278 due to a TGA stop codon (nucleotides 835–837; Supplementary Figure S1). The wild type *cirA* coding sequence has a tandem repeat of the 7-mer sequence CTGAAAC beginning at nucleotides 1,339 and 1,346; in pre-outbreak isolate SX439, a third C**TGA**AAC sequence was identified beginning at nucleotide 1,353 that extends the wild type 14-mer to a 21-mer, resulting in the introduction of a stop codon (bold) and premature truncation to 451 amino acids compared to full-length CirA containing 663 amino acids (Supplementary Figure S1). The wild type *cirA* coding region repeats an imperfect 16 bp sequence (CAAAA**T/C**CTGAAAGATG) starting at nucleotides 124 and 193 with single nucleotide differences at positions 129 (T) and 198 (C); a deletion of 69 nucleotides (AA 47 to 69) was identified in the *cirA* coding region of pre-outbreak isolate SX440 due to a recombinational event between the two imperfect 16 bp repeat sequences. Thus, three different mutations were identified in the *cirA* gene of the 10 *S*. Reading isolates from the FSIS collection, and all five of the outbreak isolates contained the same premature truncation at AA278.

### 3.2 Confirmation of genetic differences in *Salmonella enterica* serovar Reading sequences from the CDC human isolate collection and the NCBI pathogen detection database

As relevant genomic differences were identified between the pre-outbreak and outbreak *S*. Reading isolates associated with turkeys/turkey products from the FSIS collection (absence of *uidA*, *pgdA*, and phage-like regions and truncation in the *cirA* gene in the outbreak isolates), the occurrence of the genetic differences was examined in 75 *S*. Reading human isolates obtained from the CDC with isolation dates ranging from 1996 to 2019 (Supplementary Tables S2–S4). Of the 41 *S*. Reading isolates collected before or in 2017, the phage-like region was present in 16 isolates (Table 2A; Supplementary Figure S2). In contrast, the presence of the phage-like region was significantly lower (*p* = 0.009) in CDC isolates collected after 2017, with only four of 34 isolates containing the ~28 kb region. The presence of the *uidA* and *pgdA* genes was also significantly lower (*p* < 0.001) in *S*. Reading isolated from humans after 2017; 81% (33/41) and 90% (37/41) of the CDC *S*. Reading isolates collected before or in 2017 contained the *uidA* and *pgdA* genes, respectively, but only 24% (8/34) of the isolates collected after 2017 harbored these genes. A similar result was observed for the *cirA* gene in *S*. Reading isolated more recently; truncation in the CirA protein was significantly higher (*p* < 0.001) in isolates collected after 2017 compared to before or in 2017 (Table 2A; Supplementary Figure S1).

**Table 2:** Genetic differences identified in *Salmonella enterica* serovar Reading sequences from the CDC human isolate collection **(A)** and from the NCBI pathogen detection database **(B)**.

(A) CDC human isolate collection								
Features	Before/in 2017		After 2017		Fisher exact test After 2017 vs. Before 2017			
	Present (%)	Absent (%)	Present (%)	Absent (%)	Odds ratio	Conf.low	Conf.high	*p* value
*uidA*	33 (80.5)	8 (19.5)	8 (23.5)	26 (76.5)	0.078	0.021	0.253	8.02E−07
*pgdA*	37 (90.2)	4 (9.8)	8 (23.5)	26 (76.5)	0.036	0.007	0.139	2.77E−09
Phage-like	16 (39)	25 (61)	4 (11.8)	30 (88.2)	0.213	0.046	0.773	9.28E−03
	**Full (%)**	**Truncated (%)**	**Full (%)**	**Truncated (%)**				
*cirA*	39 (95.1)	2 (4.9)	9 (26.5)	25 (73.5)	0.02	0.002	0.102	4.30E−10
(B) NCBI pathogen detection database								
*uidA*	475 (65.8)	247 (34.2)	309 (17.8)	1,430 (82.2)	0.112	0.092	0.137	4.83E−116
*pgdA*	576 (79.8)	146 (20.2)	501 (28.8)	1,238 (71.2)	0.103	0.082	0.127	4.34E−123
Phage-like	317 (43.9)	405 (56.1)	191 (11.0)	1,548 (89.0)	0.158	0.127	0.196	1.09E−69
	**Full (%)**	**Truncated (%)**	**Full (%)**	**Truncated (%)**				
*cirA*	593 (82.1)	129 (17.9)	490 (28.2)	1,249 (71.8)	0.085	0.068	0.107	8.37E−139

The four distinct genetic differences were further investigated in 2,461 *S*. Reading assemblies in the NCBI Pathogen Detection database (PDD; Supplementary Table S5). As observed from the FSIS and CDC isolates, a significantly greater number of *S*. Reading sequences collected before or in 2017 contained *uidA*, *pgdA*, the phage-like region, and full-length CirA protein compared to *S*. Reading sequences in the PDD collected after 2017 (Table 2B).

### 3.3 Phenotypic verification of genomic differences between *Salmonella enterica* serovar Reading isolates

Phenotypic analyses of the *cirA* and *uidA* gene functions were conducted to confirm the genetic variations observed between the pre-outbreak and outbreak isolates. CirA is a siderophore receptor for iron acquisition in *Salmonella* (Hantke, 1990), but it also serves as a receptor for a pore-forming toxin called colicin, specifically Col1b (Cascales et al., 2007). Colicin, produced as a competitive exclusion mechanism by some bacteria, including certain strains of *Escherichia coli* (*E. coli*), attaches to and kills bacteria with a CirA receptor (Weaver et al., 1981). To determine the sensitivity of the *S*. Reading isolates to the toxin, a colicin sensitivity assay was performed. Compared to the outbreak isolates (SX454-458), the pre-outbreak isolates with a full-length *cirA* gene (SX451-453) had greater sensitivity to Col1b as observed by larger zones of growth inhibition (Table 3). The deletional event within the *cirA* gene introducing a translational frame shift and premature stop codon in the outbreak isolates resulted in reduced susceptibility to Col1b, as no zones of inhibition were observed (as well as in pre-outbreak isolates SX449-450). Similarly, most of the CDC isolates of *S*. Reading from human illnesses after 2017 had reduced susceptibility to colicin, whereas many of the isolates before 2017 had greater sensitivity to colicin (Table 3). Disruption in the colicin receptor function of CirA in the outbreak isolates could provide a competitive advantage in environments containing antagonistic Col1b-producing bacteria, such as the intestinal tract of food animals or human hosts (Davies and Reeves, 1975). Although CirA is required for uptake of dihydroxybenzoylserine, this compound is a breakdown product of the high-affinity siderophores enterobactin and salmochelin. *Salmonella cirA* mutants retain the ability to utilize enterobactin and salmochelin for iron acquisition. This could be considered a cost–benefit compromise for c*irA* mutants due to a greater metabolic expenditure for the biosynthesis of enterobactin and salmochelin but reduced susceptibility to Col1b.

**Table 3:** Colicin and cefiderocol sensitivity.

Strain	Isolate	Isolation year	*cirA* status	Colicin avg ZOI (mm)	Cefiderocol avg ZOI (mm)
FSIS isolates					
SX449	FSIS1608505	2016	Truncated	No zone	18.25
SX450	FSIS1608664	2016	Truncated	No zone	18.25
SX451	FSIS1609312	2016	Full-Length	12.5	28.75
SX452	FSIS1609546	2016	Full-Length	15.16	30.13
SX453	FSIS1609557	2016	Full-Length	12.83	30.75
SX454	FSIS21923830	2019	**Truncated**	No zone	18.38
SX455	FSIS11919695	2019	**Truncated**	No zone	19.00
SX456	FSIS21923883	2019	**Truncated**	No zone	18.38
SX457	FSIS21923895	2019	**Truncated**	No zone	18.13
SX458	FSIS31901759	2019	**Truncated**	No zone	19.00
CDC *S.* Reading isolates					
SX462	CDC: AM00921	1996	Full-Length	16.5	25.00
SX463	CDC: AM01219	1996	Full-Length	23.33	26.75
SX464	CDC: AM06848	1999	Full-Length	26.5	27.38
SX465	CDC: AM07078	1999	Full-Length	19.33	29.50
SX466	CDC: AM13238	2002	Full-Length	15.33	25.25
SX467	CDC: AM14669	2002	Full-Length	23.67	26.00
SX468	CDC: AM25980	2006	Full-Length	22.16	29.50
SX469	CDC: AM27309	2006	Full-Length	15.83	26.75
SX471	CDC: 2014AM-1887	2014	Full-Length	14.67	27.00
SX 472	CDC: 2014AM-2223	2014	Full-Length	13.83	27.50
SX 473	CDC: 2016AM-0239	2016	Truncated	No zone	18.25
SX 474	CDC: 2016AM-1538	2016	Full-Length	13.5	26.25
SX475	CDC: 2016AM-1963	2016	Truncated	No zone	17.13
SX 476	CDC: 2016AM-2585	2016	Truncated	No zone	17.50
SX479	CDC: 2018AM-0247	2018	Truncated	No zone	16.50
SX480	CDC: 2018AM-0553	2018	**Truncated**	No zone	17.13
SX481	CDC: 2018AM-1657	2018	**Truncated**	No zone	17.38
SX 482	CDC: 2018AM-1905	2018	**Truncated**	No zone	19.00
SX483	CDC: 2018AM-2511	2018	**Truncated**	No zone	16.88
SX484	CDC: 2018AM-3217	2018	**Truncated**	No zone	17.38
SX485	CDC: 2018AM-3320	2018	**Truncated**	No zone	18.00
SX 486	CDC: 2018AM-3638	2018	Full-Length	13.83	26.75
SX487	CDC: 2018AM-3686	2018	**Truncated**	No zone	17.25
SX488	CDC: 2018K-0324	2018	**Truncated**	No zone	18.00
SX 489	CDC: 2019K-0247	2018	**Truncated**	No zone	18.25
SX490	CDC: 2019AM-0413	2019	**Truncated**	No zone	17.83
SX 491	CDC: 2019AM-0414	2019	**Truncated**	No zone	18.25
*S.* Typhimurium strains					
BSX 8	χ4232				26.59
BBS 92	BSX 8 *ΔcirA*				18.75
BBS 46	BSX 8 *ΔfepA*				25.50
BBS 47	BSX 8 Δ*iroN*				25.00
BBS 83	BSX 8 *ΔfepA ΔcirA*				15.00
BBS 80	BSX 8 *ΔiroN ΔcirA*				17.75
BBS 81	BSX 8 *ΔfepA ΔiroN*				24.75

**Figure 2:**
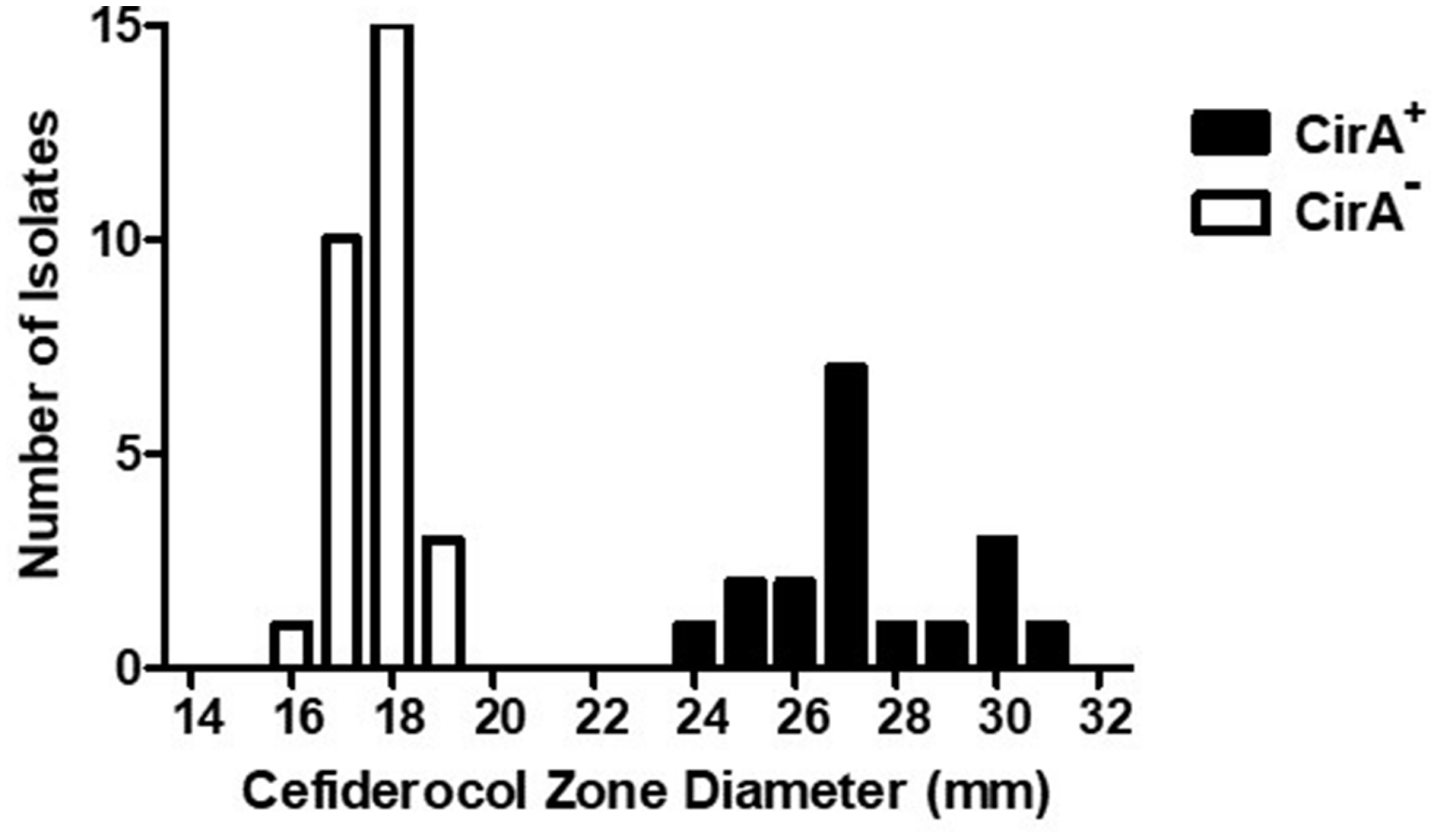
Cefiderocol zone diameters for *Salmonella enterica* serovar Reading isolates with wild type and truncated CirA. Zone of growth inhibition (mm) around a cefiderocol disk (30 μg) for *S.* Reading isolates (FSIS and CDC) with wild type (+) and truncated (−) CirA. Isolates were grown on a Mueller-Hinton agar plate for 18–20 h at 37°C prior to measuring susceptibility to cefiderocol.

An additional phenotypic advantage of the CirA truncation could be reduced sensitivity to the antimicrobial cefiderocol. Cefiderocol is a catechol-type siderophore conjugated to a cephalosporin (beta-lactam) moiety with antimicrobial activity against aerobic Gram-negative bacteria (Ito et al., 2018; McCreary et al., 2021). In the United States, cefiderocol is approved for human treatment of complicated urinary tract infections, hospital-acquired pneumonia, and ventilator-associated bacterial pneumonia. Due to its siderophore structure, cefiderocol binds siderophore receptor proteins (SRPs) in the outer membrane and is transported into the periplasmic space, where it interacts with penicillin-binding proteins to inhibit bacterial cell wall biosynthesis. Mutations in *cirA*, encoding an SRP, decrease *E. coli* susceptibility to cefiderocol (Ito et al., 2018), but mutations that alter antimicrobial susceptibility in *Salmonella* have not been described. Due to *E. coli cirA* mutants having reduced antibiotic sensitivity and outbreak-associated isolates of *S*. Reading having an increased frequency of CirA truncation, cefiderocol susceptibility assays were performed with pre-outbreak and outbreak *S*. Reading. Isolates predicted by genomic sequencing to encode an entire CirA (and therefore would be susceptible to cefiderocol) had a cefiderocol zone of inhibition (ZOI) that ranged from 24 to 31 mm with an average of 27, whereas isolates with a predicted CirA truncation had a cefiderocol ZOI ranging from 16 to 19 mm with an average of 18 (Figure 2; Table 3). For *S*. Reading isolates from turkeys/turkey products (FSIS), the average cefiderocol ZOI for pre-outbreak isolates was larger (greater susceptibility) compared to outbreak isolates, and *S*. Reading isolates from humans (CDC) had a larger cefiderocol ZOI before 2017 than after 2017. This analysis demonstrates that cefiderocol susceptibility may be used as a phenotypic assay to differentiate isolates with and without a functional CirA protein, though further validation for set limits is warranted.

*Salmonella* serovars encode multiple SRPs, including CirA, FepA, and IroN. FepA SRP binds enterobactin, and the IroN SRP binds salmochelin, a glucosylated form of enterobactin (Fischbach et al., 2005). Breakdown products of salmochelin and enterobactin bind to the CirA SRP (Hantke, 1990). Due to IroN, FepA, and CirA being catecholate SRPs, cefiderocol susceptibility was determined for *Salmonella enterica* serovar Typhimurium (*S.* Typhimurium) strains containing individual *iroN*, *fepA*, and *cirA* mutations or in combination (Bearson et al., 2008). Similar to serovar Reading, a *cirA* mutation in strain BBS 92 decreased *S.* Typhimurium susceptibility to cefiderocol compared to wild type strain BSX 8 (Table 3). Further modest decreases in cefiderocol susceptibility for *S.* Typhimurium occurred by combining a *cirA* mutation with mutations in either the *fepA* or *iroN* genes. Only slight reductions in cefiderocol susceptibility were observed for single mutations in the *fepA* and *iroN* genes or in a *fepA*-*iroN* double mutant of *S.* Typhimurium. The data suggest that the hierarchy for *S.* Typhimurium cefiderocol susceptibility due to SRPs is CirA> > FepA > IroN. To the best of our knowledge, this is the first description of gene mutations involved in decreased susceptibility to cefiderocol in the genus *Salmonella*.

Glucuronides can be utilized by some bacteria as a carbon source (Little et al., 2018), and β-D-glucuronidase encoded by the *uidA* gene may enhance the utilization of glucuronides by *S.* Reading isolates in certain environments. As the pre-outbreak and outbreak isolates differed in *uidA* genetic composition, Biolog Phenotype Microarray microplate PM2 containing β-methyl-D-glucuronic acid was employed to compare the utilization of the carbon source by the pre-outbreak (*uidA*^+^) and outbreak (*uidA*^−^) isolates from turkeys/turkey products. Utilization of β-methyl-D-glucuronic acid (measured via microbial respiration) was significantly greater (*p* = 0.0105) for the pre-outbreak isolates compared to the outbreak isolates (Supplementary Figure S3). However, pre-outbreak isolate SX442 did not have a phenotypic increase in respiration in response to β-methyl-D-glucuronic acid. Snippy analysis within the pre-outbreak isolates detected a single nucleotide deletion in the intergenic region 135 base pairs upstream of the *uidA* start codon in FSIS pre-outbreak isolate SX442 that may impact operon expression, resulting in a lack of phenotypic utilization of β-methyl-D-glucuronic acid (data not shown).

### 3.4 Colonization potential of turkeys with pre-outbreak and outbreak isolates of *Salmonella enterica* serovar Reading from turkeys/turkey products

**Figure 3:**
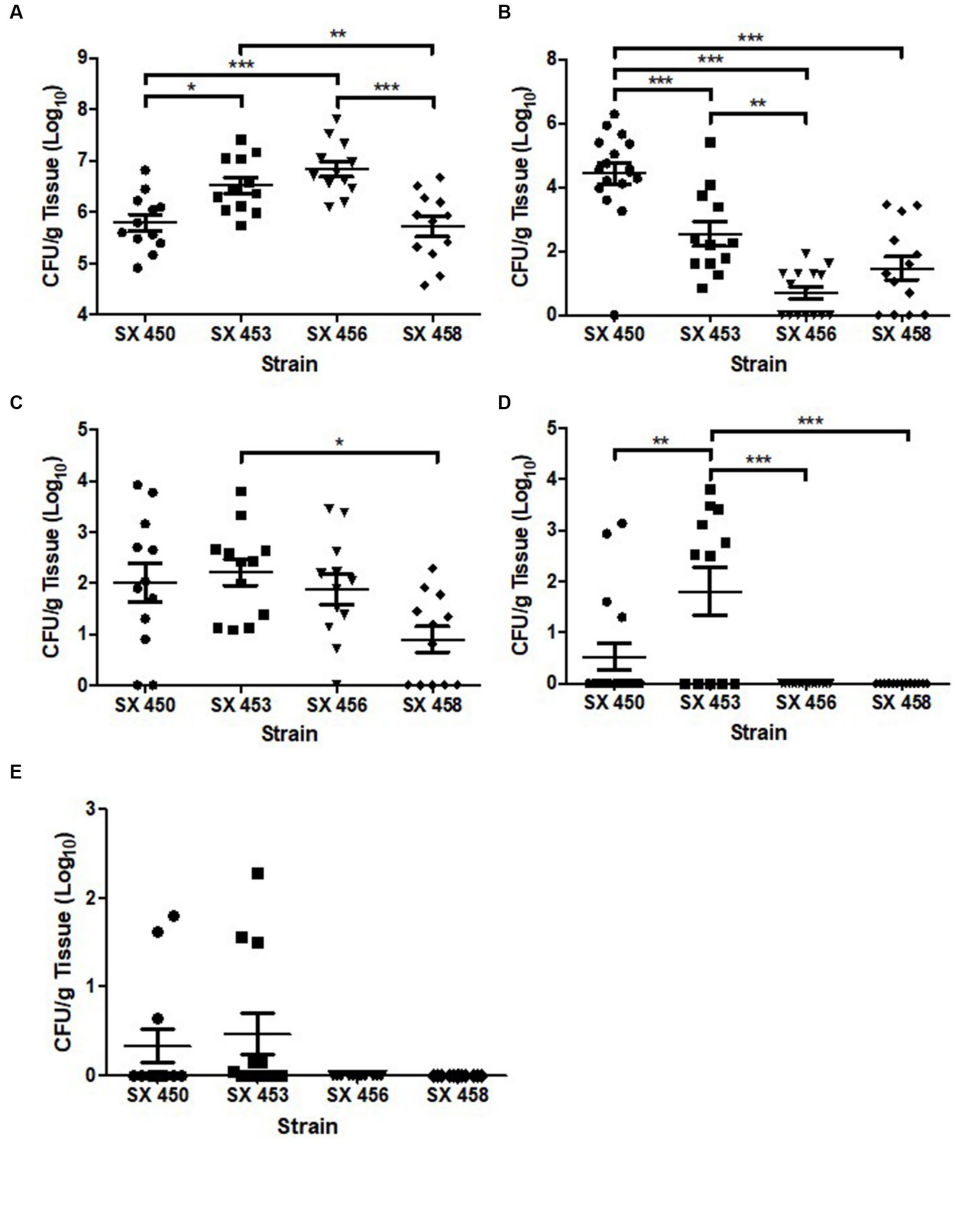
*Salmonella enterica* serovar Reading colonization of turkey cecum, bursa of Fabricius, and spleen at 1-week and 5-weeks post-inoculation. At 1 day of age, turkey poults were inoculated with 1 × 10^4^ CFU of pre-outbreak (SX450 and SX453) and outbreak (SX456 and SX458) isolates of *Salmonella* Reading in four separate ABSL-2 isolation rooms. On day 7 following *S.* Reading inoculation, 12 poults from each group were euthanized, and the remaining turkeys were euthanized at 5-weeks post-inoculation. Following euthanasia, tissues [cecum (**A**, week 1; **B**, week 5), bursa of Fabricius (**C**, week 1; **D**, week 5), and spleen (**E**, week 1)] were harvested for quantitative and qualitative bacteriology to determine tissue colonization by *S*. Reading. Each point indicates an individual bird, horizontal bars denote the mean for each group, and error bars designate the standard error of the mean (SEM). Statistically significant differences between groups are denoted by ^*^p < 0.05, ^**^p < 0.01, and ^***^p < 0.001.

A turkey challenge study was performed to compare the colonization capability of two pre-outbreak (SX450 and SX453) and two outbreak (SX456 and SX458) *S*. Reading isolates from turkeys/turkey products. Following the challenge of 1-day-old poults in separate isolation rooms with 1 × 10^4^ CFU of *S*. Reading, quantitative and qualitative bacteriology measured the colonization loads in 10–12 turkeys during the acute (1-week post-inoculation) and persistent (5-week post-inoculation) phases of colonization in the cecum (intestinal tract), bursa of Fabricius (primary lymphoid organ), and spleen (systemic dissemination). For the cecum, the four *S*. Reading isolates quantitatively colonized all birds in the range of 1 × 10^4–8^ CFU/g at 7 dpi, with significant differences presented in Figure 3. At 35 dpi, significantly greater colonization of SX450 persisted in the cecum compared to the other three isolates. For the bursa of Fabricius, similar colonization rates were observed between the four *S*. Reading isolates at 7 dpi. At 35 dpi, all turkeys challenged with the two outbreak *S*. Reading isolates tested negative for *Salmonella* in the bursa of Fabricius, whereas four (SX450) and seven (SX453) turkeys challenged with the pre-outbreak isolates were positive for *Salmonella* at up to 1 × 10^4^ CFU/g. Systemic dissemination to the spleen was not observed at 7 dpi for the two outbreak *S*. Reading isolates, while three (SX450) and six (SX453) turkeys challenged with the pre-outbreak isolates tested positive for *Salmonella* (although not significantly different); all spleens were negative for *Salmonella* at 5-week post-inoculation regardless of the inoculation isolate. Thus, a dramatic difference in acute (1-week post-inoculation) colonization was not observed between the *S*. Reading isolates, but higher colonization levels detected at 5-week post-inoculation suggest greater persistence potential for the pre-outbreak isolates.

## 4 Discussion

In the *S.* Reading isolates from turkeys/turkey products, genetic variation was observed between the isolates before the defined outbreak period (pre-outbreak) compared to isolates during the outbreak, and a similar pattern was also detected in the human isolates of *S.* Reading. Overall, a comparable genome reduction pattern, partially due to predicted recombinational events, was generally observed in both the turkey and human isolates of *S*. Reading during the outbreak period, and the genetic differences were present in genes that could influence *Salmonella* colonization, fitness, and/or virulence.

A WGS comparison of 988 *S*. Reading isolates from humans, meat, and live food animals in the U.S. identified three major clades of *S*. Reading, with one highly adapted turkey clade (Miller et al., 2020). The isolates within the turkey clade clustered into three subclades described as historical (1999–2008), contemporary (2009–2019, with most from 2009 to 2016), and emergent (2017–2019) isolates. Similar genetic features described for the emergent subclade were observed in isolates of this study recovered from the outbreak-associated period, including deletion of the *uidA* and *pgdA* genes as well as premature truncation of CirA due to a translational frameshift. Miller et al. also describe an internal deletion of AA 47–69 in CirA for a subset of contemporary isolates, and we identified the same genetic mutation in pre-outbreak isolate SX440 and six CDC isolates due to a recombinational event between two 16-mer duplications with a single mismatch.

In an analysis of *Salmonella* genomic content for 98 serovars, including *S*. Reading, den Bakker et al. describe serovars with and without β-glucuronide utilization genes (Den Bakker et al., 2011). Serovars with the β-glucuronidase operon consistently have three SNPs that differentiate them from serovars that do not contain genes for the utilization of β-glucuronides. Genome sequence analysis of the FSIS *S.* Reading pre-outbreak and outbreak isolates indicated that all 10 isolates contained the three SNPs associated with the presence of β-glucuronide utilization genes, even though the five outbreak isolates did not harbor the genes for β-glucuronide utilization. The SNP genotype for *S.* Reading pre-outbreak and outbreak isolates supports the process of genome reduction within *S*. Reading rather than the acquisition of the β-glucuronidase operon in a subset of isolates.

The lack of utilization of β-methyl-D-glucuronic acid by the outbreak isolates of *S.* Reading was predicted as genomic analysis identified the absence of an ~5.4 kb region containing the *uidA* and *pgdA* genes. den Bakker et al. demonstrate that only 27 of the 98 serovars (28%) investigated contain genes encoding β-glucuronidase activity. Furthermore, numerous *Salmonella* serovars that are typically included in the top 10 lists of serovars isolated from human foodborne disease (Enteritidis, Typhimurium, Newport, Infantis, Paratyphi A, and Saintpaul) and turkeys (Infantis, Hadar, Typhimurium, Uganda, Senftenberg, Agona, Newport, I 4,[5],12:i:-, and Heidelberg) do not contain genes encoding β-glucuronidase activity. Only serovars Javiana and Montevideo of the top 10 human-illness-associated *Salmonella* serovars contain genes for β-glucuronidase activity; similarly, Reading and Schwarzengrund are the top 10 serovars from turkeys with β-glucuronidase activity. Therefore, the presence of genes encoding β-glucuronidase activity in frequently isolated *Salmonella* serovars appears to be a minority, especially among serovars that have the greatest prevalence in turkey colonization and human illness. Although β-glucuronidase activity may expand bacterial carbon utilization in the gastrointestinal tract, it does not appear to be a pre-requisite for colonization or pathogenesis, and it is unclear how the loss of function could have contributed to the outbreak status of *S.* Reading.

The primary function of CirA in *Salmonella* is iron acquisition as a siderophore receptor for transport of dihydroxybenzoylserine, a breakdown product of the high-affinity siderophores salmochelin and enterochelin (Hantke, 1990). However, colicin Ia/b, a channel-forming bactericidal protein, also utilizes a functional CirA as its receptor, resulting in bacterial vulnerability (Weaver et al., 1981; Cascales et al., 2007). The colicin sensitivity assay demonstrated that *S.* Reading isolates with a truncated CirA had decreased susceptibility to colicin. Genomic analysis identified three different types of *cirA* mutations within the FSIS and CDC isolate collections that resulted in reduced sensitivity to colicin, suggesting that *cirA* mutations may result in an advantageous phenotypic outcome. For example, disruption in the colicin receptor function of CirA in *S.* Reading outbreak isolates could provide a competitive advantage in environments containing antagonistic Col1b-producing bacteria, such as the intestinal tract of food animals or human hosts. However, the turkey inoculation study indicated that pre-outbreak isolates of *S.* Reading with a full-length *cirA* gene had higher colonization levels at 5-week post-inoculation compared to outbreak-associated isolates, suggesting greater persistence potential for the pre-outbreak isolates. Colicin production by the cecal microbiome of the turkeys in the *S*. Reading challenge study was not determined, but based on the colicin sensitivity data, we would predict that if colicin-producing bacteria were present in the cecum, the colonization potential of the pre-outbreak and outbreak-associated isolates may have differed, with colonization favoring the post-outbreak isolates. Even though an impact on turkey colonization was not identified in the present study, the ability of *S*. Reading to survive, colonize, and/or persist in other environments, such as the human intestinal tract, could be influenced by the colicin receptor status.

In addition to colicin sensitivity assays, cefiderocol susceptibility may be used as a phenotypic assay to differentiate *Salmonella* isolates with and without a functional CirA protein. The cefiderocol disk diffusion assay is based on routine antimicrobial susceptibility testing, is less intensive, and the results are available in a shorter time period compared to the determination of colicin sensitivity. The siderophore receptor mutants of *S.* Typhimurium indicate that CirA is the primary receptor for cefiderocol, while both FepA and IroN provide modest transport. Inactivation of either FepA or IroN further reduced the susceptibility of *Salmonella* to cefiderocol when a functional CirA was absent.

Outbreak-associated *S.* Reading isolates contained additional SNPs compared to the pre-outbreak isolates, but phenotypic investigations were not performed to evaluate if these SNPs may alter bacterial physiology and metabolism or modify host colonization and virulence phenotypes. Furthermore, many of the identified genetic differences involved genes encoding hypothetical proteins that could potentially play a role in *Salmonella* fitness in the host. Further investigation and elucidation of the role of these genetic alterations in the emergence of outbreak-associated *S.* Reading is warranted.

The indistinguishable PFGE pattern (CDC, 2019; Hassan et al., 2019) and the timeline described for the emergence of the novel clonal group of *S*. Reading (Miller et al., 2020) suggest that outbreak *S*. Reading originated from a common source and disseminated swiftly through the turkey industry regardless of geographical region, production facility, or company (Miller et al., 2020). Miller et al. proposed that the massive depopulation and expeditious repopulation of turkey farms due to the devastating 2015 highly pathogenic avian influenza outbreak provided an opportunity for rapid clonal expansion of the emergent clade of *S*. Reading via the breeder turkey flock. Our phenotypic data indicated that the emergent *S*. Reading associated with the foodborne outbreak has decreased susceptibility to colicin toxin; thus, one could hypothesize that if the turkey breeder flocks contained a colicin-producing bacterium in their intestinal tract, selective pressure for *S*. Reading with a truncated CirA receptor could have occurred. The current study illustrates the usefulness of paired genomic and phenotypic analyses in outbreak investigations of the foodborne pathogen *Salmonella* involving food animal production.

## Data availability statement

The datasets presented in this study can be found in online repositories. The names of the repository/repositories and accession number(s) can be found in the article/Supplementary material.

## Ethics statement

The animal study was approved by USDA, ARS, National Animal Disease Center Animal Care and Use Committee. The study was conducted in accordance with the local legislation and institutional requirements.

## Author contributions

SS: Data curation, Formal analysis, Investigation, Methodology, Software, Visualization, Writing – review & editing. BB: Conceptualization, Data curation, Formal analysis, Investigation, Methodology, Software, Visualization, Writing – original draft, Writing – review & editing. JT: Data curation, Formal analysis, Investigation, Writing – review & editing. DN: Data curation, Software, Writing – review & editing. TL: Conceptualization, Investigation, Writing – review & editing. SB: Conceptualization, Data curation, Formal analysis, Investigation, Methodology, Project administration, Resources, Supervision, Validation, Visualization, Writing – original draft, Writing – review & editing.
