# Supplementary figures and images for: Genomic and phenotypic characterization of multidrug-resistant Salmonella enterica serovar Reading isolates involved in a turkey-associated foodborne outbreak
Source: Front Microbiol. 2024 Jan 18;14:1304029. doi: 10.3389/fmicb.2023.1304029 (PMC10830755; doi:10.3389/fmicb.2023.1304029)

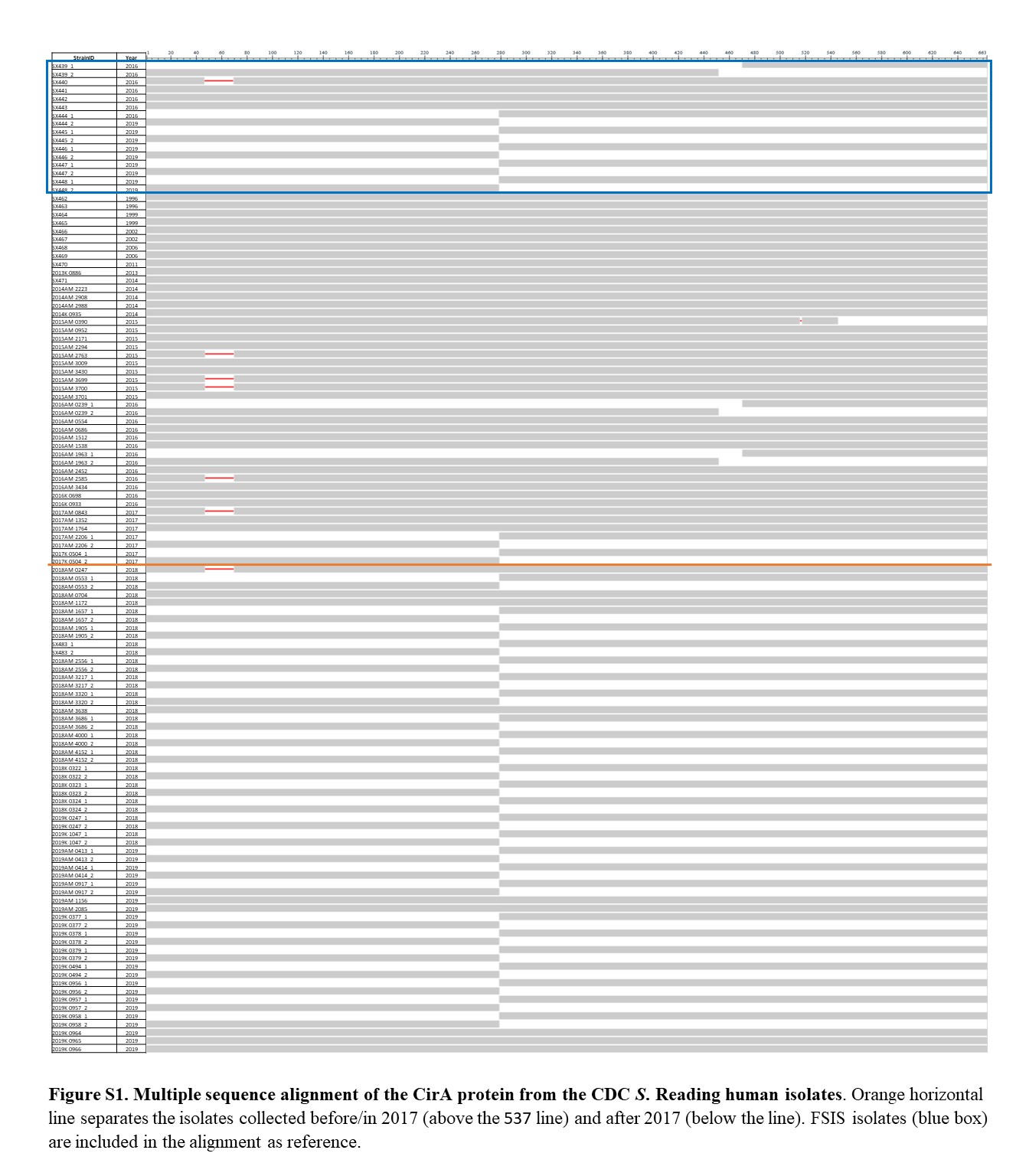

Supplement: Supplementary file 1 [file Image_1.jpg]

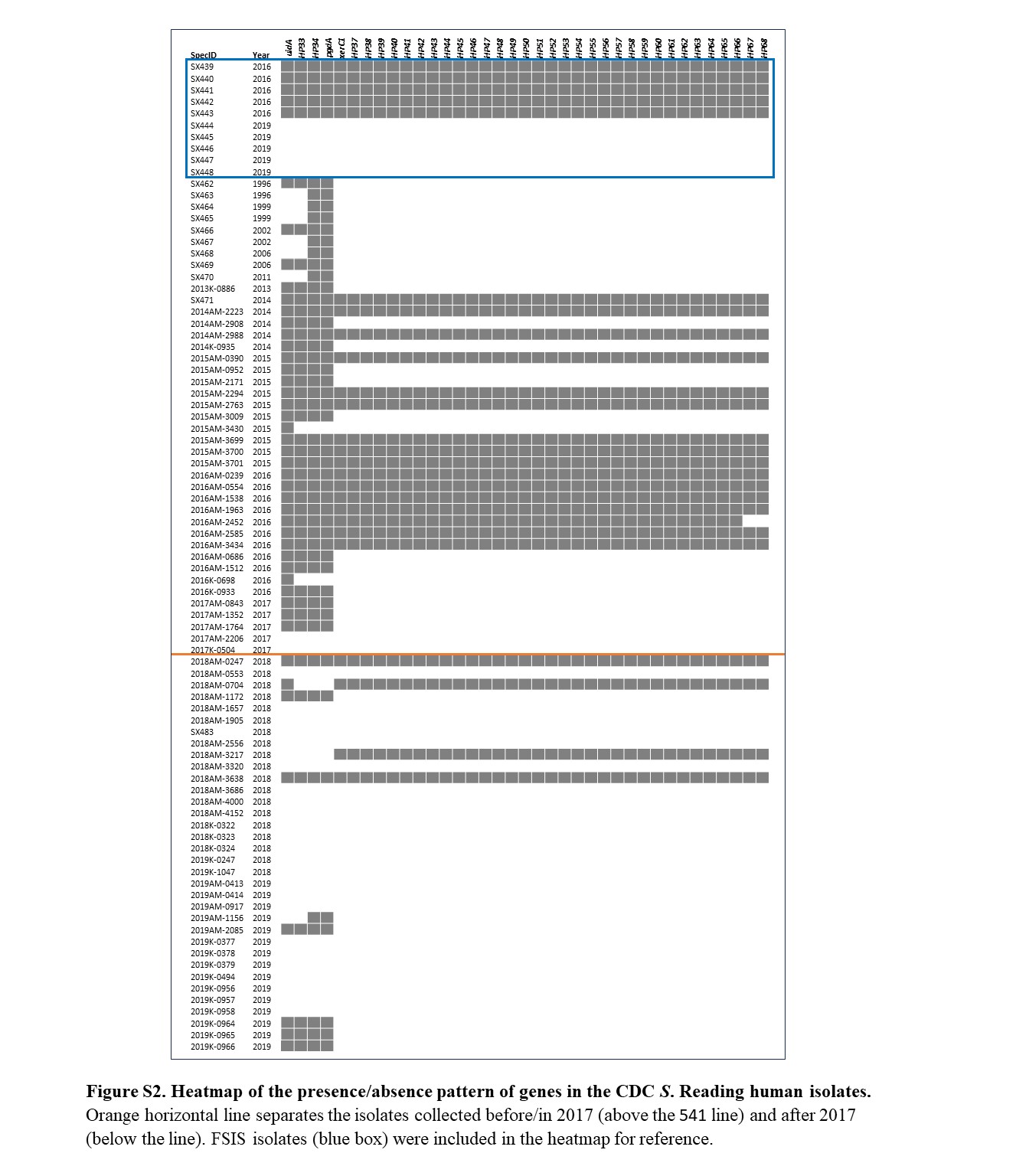

Supplement: Supplementary file 2 [file Image_2.jpg]

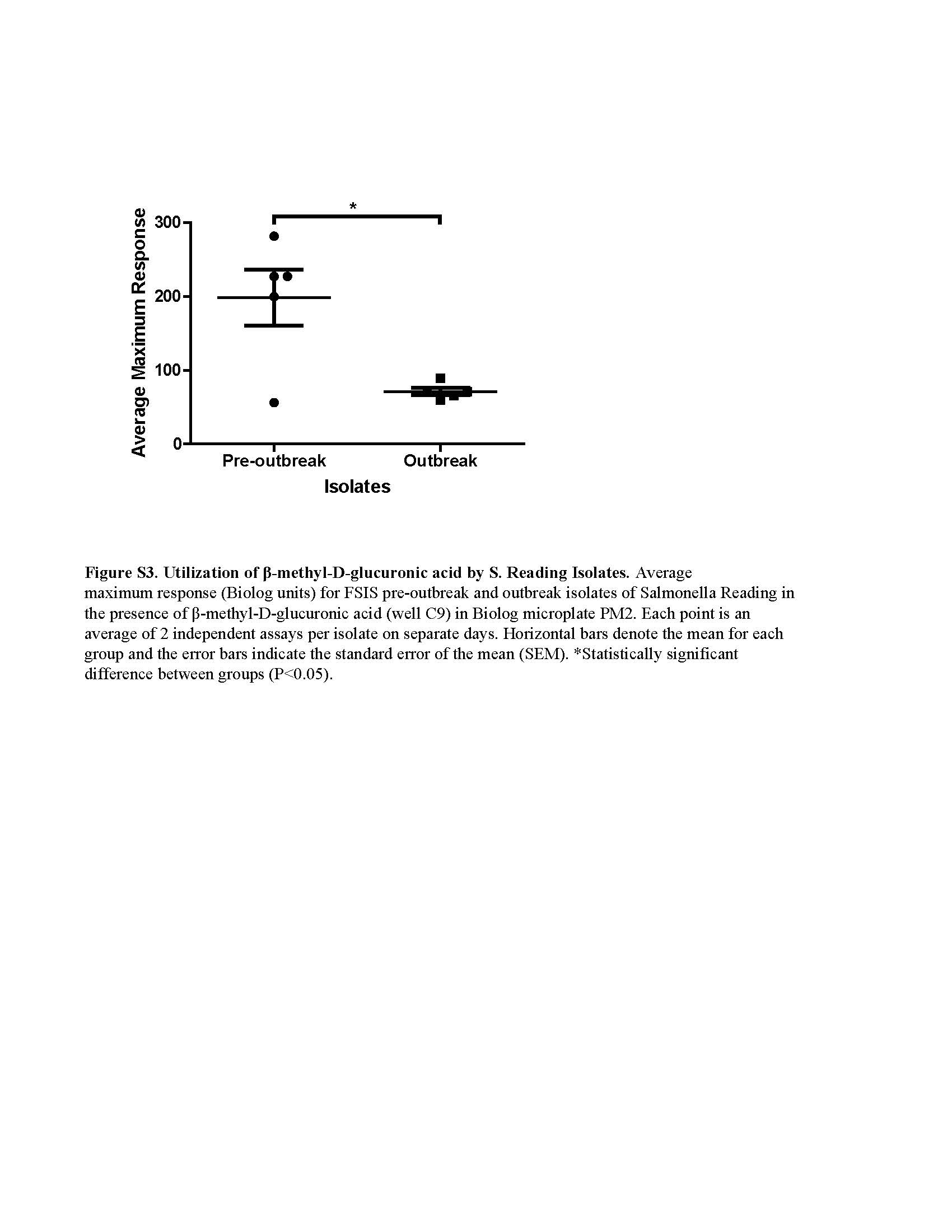

Supplement: Supplementary file 3 [file Image_3.jpg]
